# Shifting transmission risk for malaria in Africa with climate change: a framework for planning and intervention

**DOI:** 10.1186/s12936-020-03224-6

**Published:** 2020-05-01

**Authors:** Sadie J. Ryan, Catherine A. Lippi, Fernanda Zermoglio

**Affiliations:** 1 Emerging Pathogens Institute, University of Florida, Gainesville, FL USA; 2 Department of Geography, University of Florida, Gainesville, FL USA; 3 College of Agriculture, Engineering, and Science, University of KwaZulu-Natal, Durban, South Africa; 4 Chemonics International, Washington, DC, USA

**Keywords:** Malaria, Africa, *Anopheles*, Temperature, Climate change

## Abstract

**Background:**

Malaria continues to be a disease of massive burden in Africa, and the public health resources targeted at surveillance, prevention, control, and intervention comprise large outlays of expense. Malaria transmission is largely constrained by the suitability of the climate for *Anopheles* mosquitoes and *Plasmodium* parasite development. Thus, as climate changes, shifts in geographic locations suitable for transmission, and differing lengths of seasons of suitability will occur, which will require changes in the types and amounts of resources.

**Methods:**

The shifting geographic risk of malaria transmission was mapped, in context of changing seasonality (i.e. endemic to epidemic, and vice versa), and the number of people affected. A published temperature-dependent model of malaria transmission suitability was applied to continental gridded climate data for multiple future AR5 climate model projections. The resulting outcomes were aligned with programmatic needs to provide summaries at national and regional scales for the African continent. Model outcomes were combined with population projections to estimate the population at risk at three points in the future, 2030, 2050, and 2080, under two scenarios of greenhouse gas emissions (RCP4.5 and RCP8.5).

**Results:**

Estimated geographic shifts in endemic and seasonal suitability for malaria transmission were observed across all future scenarios of climate change. The worst-case regional scenario (RCP8.5) of climate change predicted an additional 75.9 million people at risk from endemic (10-12 months) exposure to malaria transmission in Eastern and Southern Africa by the year 2080, with the greatest population at risk in Eastern Africa. Despite a predominance of reduction in season length, a net gain of 51.3 million additional people is predicted be put at some level of risk in Western Africa by midcentury.

**Conclusions:**

This study provides an updated view of potential malaria geographic shifts in Africa under climate change for the more recent climate model projections (AR5), and a tool for aligning findings with programmatic needs at key scales for decision-makers. In describing shifting seasonality, it was possible to capture transitions between endemic and epidemic risk areas, to facilitate the planning for interventions aimed at year-round risk versus anticipatory surveillance and rapid response to potential outbreak locations.

## Background

Malaria causes an estimated 435,000 deaths per year, with the majority of cases occurring in sub-Saharan Africa, affecting children under 5 disproportionately [1]. Recent advances in reducing case burdens in sub-Saharan Africa through bed net distribution, household level spraying, and rapid clinical diagnostic and treatment responses appeared to slow down in 2017 and 2018, leaving reduction, and eradication goals unmet, and an estimated 219 million cases in 2018 [1]. The World Health Organization reported that for 10 high burden African countries, there was an increase of 3.5 million cases in 2017 over the prior year. This stall in reduction was largely attributed to a stall in investments in global responses to malaria. The U.S. remained the single largest international donor in 2017, contributing $1.2 billion (39% of the overall investment); it is projected that roughly $6.6 billion annually by 2020 will be needed for the global malaria strategy, underscoring the importance of knowing how much and where to invest.

Geospatial modelling approaches provide a flexible framework in which to explore possible future scenarios of malaria risk as a function of changing climate [2]. Mordecai et al. introduced a mechanistic nonlinear physiological temperature-driven malaria transmission suitability model in 2013, via incorporating temperature dependent traits of both the mosquito and parasite, based on laboratory data [3]. This demonstrated that transmissibility of malaria is constrained between 17 and 34 ℃, which will therefore limit the spatial distribution of malaria on the landscape. In addition, this model updated the optimum temperature for malaria transmission from 31 ℃ to 25 ℃, and the model was well validated using 40 years of field observation data matched to specific location month and temperature [3]. Temperature has also been shown to be an important predictor of incidence in many locations [4], and the potential effects of climate-induced temperature shifts as an impact on intervention and vector control efforts have been noted [5]. In previous work, the top quantile of predicted transmission suitability from the Mordecai et al. model, that is, the top 25% of the transmission or *R*_0_ curve, was found to best capture spatial and seasonal risk for Africa, from independent models of malaria risk prediction, based on statistical models of spatial case data from the Mapping Malaria Risk in Africa (MARA) and Malaria Atlas Project (MAP) projects [2, 6–8].

Climate change threatens to the alter the nature of future malaria exposure across Sub-Saharan Africa [2, 6, 7]. Many countries with a high burden of malaria now have weak surveillance systems and are not well positioned to assess disease distribution and trends, making it difficult to optimize responses and respond to outbreaks [9]. To date, knowledge on how climate driven changes in malaria risk will manifest at regional and national scales is limited, though such knowledge is critical to designing responses. Changes in both the areas and populations exposed to malaria risk will necessitate adaptive responses to address them. To inform these responses, six scenarios of changing suitability, aligned to potential management strategies to address the changing risks, were explored. This provides an updated view of climate-driven malaria shifts in Africa from the 2015 mapping paper by Ryan et al. [2], using the newer IPCC AR5 climate change scenario framework, explicitly defining season length to align with policy language, and including a sub-continental approach, aligning changes to regional scale planning.

The goals of this study were to (1) identify new areas that will emerge as suitable for malaria transmission under different scenarios of change; (2) identify areas that may experience reductions in transmission suitability season length; and (3) provide an estimate of the human population at risk under each scenario. These are presented in the language of malaria seasonality risk, to align with surveillance and intervention targeting goals, and summarized as regional scale outcomes, broadly aligned with USAID’s planning scales, as the parent aid organization of much of the US investment in the global malaria strategy.

## Methods

### Malaria transmission

The model for temperature-dependent malaria transmission presented in Mordecai et al. [3] used this expression for *R*_*0*_, the basic reproductive rate of the disease, in order to account for the fitting of these rates to laboratory measurements:

$$R_{0} = \sqrt {\frac{{a^{2} bcmp^{T} }}{{\left( { - \ln p} \right)r}}} .$$

The temperature-dependent parameters are the mosquito biting rate (a), vector competence (b*c), mosquito density (m), the mosquito survival rate (p), and the parasite’s extrinsic incubation period (T), all of which are measurable empirical parameters.

The model incorporated temperature response curves fit for the mosquito species *Anopheles gambiae* and the malaria pathogen *Plasmodium falciparum*, with additional information used for related *Anopheles* and *Plasmodium* species. Transmission, *R*_0_ was scaled from 0 to 1, to describe relative transmission suitability across the range of temperature. In the paper by Ryan et al. [2], the top quantile (upper 25 percent) of the curve was selected to represent the range of temperatures in which transmission suitability is expected. This conservative measure of the overall temperature curve corresponds to existing maps of ongoing transmission under current temperatures [2]; this is a simple temperature range estimate for transmission (22.9–27.8 ℃), and is presented as such.

### Climate data

Current temperature data is represented by globally gridded 5 arc-minute WorldClim (version 1) monthly mean temperature data [10]. This represents a long-term average, or baseline, which has been used to project future climate scenarios and, therefore, serves as the baseline.

General Circulation Models (GCMs) are the primary source of information about potential future climate. GCMs comprise simplified but systematically rigorous mathematical descriptions of physical and chemical processes governing climate, including the role of the atmosphere, land, oceans, and biological processes. They allow for modelling the expected climate response to increasing greenhouse gas concentrations. The direct application of GCM output to adaptation decision making, however, has been relatively limited due to GCMs’ coarse spatial resolution (100 to 500 km^2^). For strategic planning in malaria prevention and control, information is required on a much more local scale than GCMs can provide. Here, a statistically downscaled multi-model ensemble product is used for this analysis, compiled at a resolution of 5 arc-minutes (~ 10 km^2^) from 6 downscaled GCMs. The climate projection data used in this study consisted of the median value for the multimodel ensemble representing future climate, compiled from the Coupled Model Intercomparison Project (CMIP5) archive, downscaled using a Change Factor (CF) approach and sourced from Navarro-Racines et al. [11]. This ensemble approach allows exploration of the range of uncertainty across climate projections under two greenhouse gas emissions scenarios, or Representative Concentration Pathways (RCPs)–RCP 4.5 and RCP 8.5—for three future time periods: the 2030s, 2050s, and 2080s. Using an ensemble model under two RCPs creates chosen bounds on potential future mitigation strategy outcomes, but neither the range of values within the models ensembled, nor present uncertainty estimates for the climate models, was explore as that is out of scope for the current study.

### Aridity masking

*Anopheles* mosquitoes (i.e., malaria-transmitting mosquitoes) require an appropriate level of moisture in their environment to provide breeding habitat with which to complete their lifecycle. Humidity or moisture is thus another component in the climate–transmission relationship. While several models use rainfall as a predictor for malaria occurrence, it is complicated to generalize how precipitation measures, such as monthly rainfall totals, cumulative rainfall, or relative humidity, actually manifest as breeding habitat for mosquitoes at large scales [12–15].

Precipitation may not be a good indicator of standing water, and in a world of increasingly extreme precipitation events, the difference between a month’s rainfall occurring in a single day versus gradual accumulation over that month becomes more relevant. Mosquito habitat can wash away, “flushing” away eggs and disrupting the lifecycle, meaning that more rain does not necessarily translate into more habitat [16]. In addition, much of the world is subject to agricultural irrigation, redirecting precipitation in nonlinear ways at local level, or even creating piped water environments in the absence of precipitation. To generalize habitat suitability for mosquito breeding, a remotely sensed proxy is used: the normalized difference vegetation index (NDVI), which measures the photosynthetic activity of growing plant matter, on a 0-1 scale. The NDVI is thus a useful descriptor of the type of habitat conducive to *Anopheles* breeding. The threshold of “too dry” is based on prior work conducted by Suzuki et al. [17] to exclude locations where the NDVI drops below a critical minimum level for two months of the year, thereby cutting off breeding and the transmission cycle [17]. A modified version of the methods of Ryan et al. [2] was used to limit projected models to those geographic areas capable of supporting mosquito survival. Monthly NDVI values were derived from post-processed MODIS data, available from FEWS-Net (Famine Early Warning System Network) [18] and month-to-month thresholding was calculated [17]. That is, if the NDVI value for two consecutive months fall below 0.125, it is assumed that an aridity boundary is crossed, indicating that that area (pixel) is considered too arid for malaria transmission to occur. The 2016–2017 period of NDVI was chosen as an average climate year for the current decade. As NDVI cannot be projected into future scenarios, an average current aridity mask was used, which is a conservative approach.

### Population data

Global gridded population products, the Gridded Population of the World (GPW), at a 30 arc-second (~ 1 km^2^) resolution were downloaded. Population data for Africa used as input for calculating population at risk (PAR) under the various transmission scenarios were derived from the Gridded Population of the World, Version 4 (GPWv4) [19], with baseline estimates derived from 2015 GPW data, while projected future populations were extracted from the 2020 layers.

### Geospatial projections of transmission

The gridded temperature data (current and future climate scenarios, month-wise) were constrained to the temperature range of the optimal quantile of transmission, and the resulting number of months of transmission suitability in each pixel recorded for all of Africa. The aridity mask was applied, and pixels falling in masked areas were given no value.

Seasons of transmission were defined based on the numbers of months of suitability, and criteria established by MARA were followed in defining malaria transmission suitability, with very slight additional granularity to better illustrate the impact of changing climate (Table 1).

**Table 1:** Definitions of malaria transmission suitability used in summarizing areas and population at risk

Malaria suitability	Definition
Endemic	Malaria transmission suitability for 10–12 months of the year
Seasonal	Malaria transmission suitability for 7–9 months of the year
Moderate	Malaria transmission suitability for 4–6 months of the year
Marginal	Malaria transmission suitability for 1–3 months of the year

**Fig. 1:**
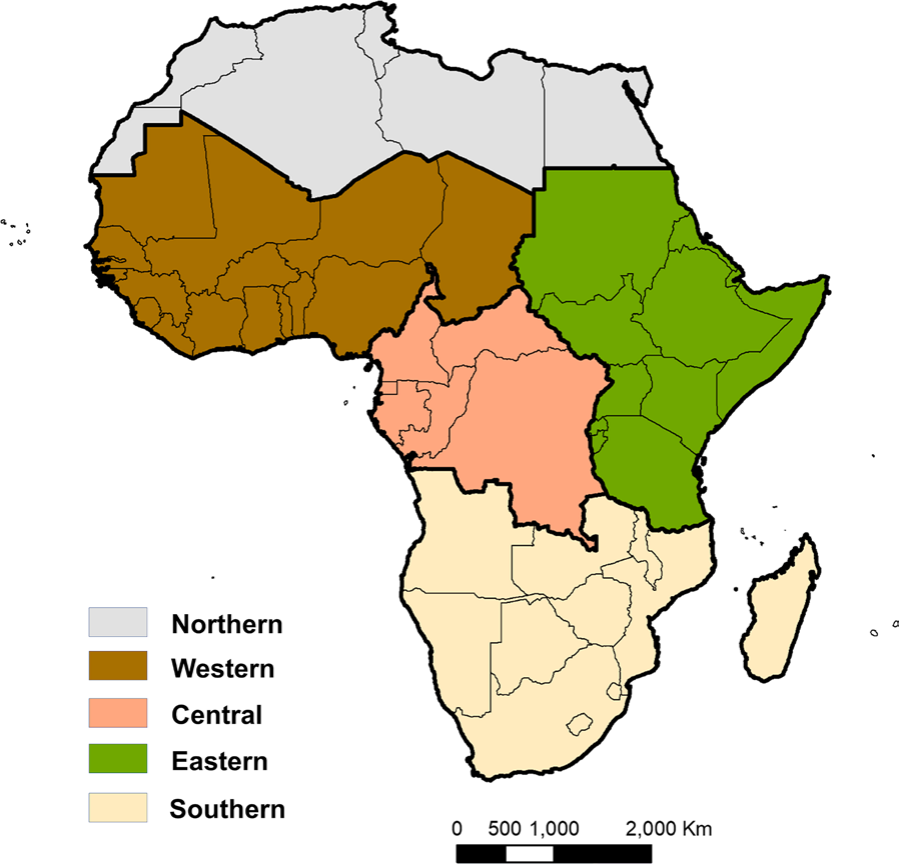
Map of the five regional definitions of Africa used in this study. Note that the Northern Africa region was excluded from analyses in this study

In order to estimate the population at risk (PAR) for each geospatial research question, the suitability data were aggregated by a factor of 10 and aligned to the climate data, such that all analyses were conducted at 5 arc-minute resolution (approximately 10 km^2^ at the equator). Population data for each scenario were summarized by region, shown in Fig. 1. Five regions of Africa were defined; these align with the policy scale, but not definition of countries for USAID’s four African regions. Eastern Africa and Central Africa were delineated to align with physical geography—while USAID defines Eastern Africa to include the Democratic Republic of Congo and Congo, and Central African Republic, Cameroon, Gabon and Equatorial Guinea are all included in the USAID West African Region, a Central Africa region was defined, comprising these countries (Fig. 1). The models are presented for four of these regions, excluding Northern Africa from this study.

All calculations and analyses were conducted in R [R version 3.3.3 2017-03-06 “Another Canoe”] using the “raster,” “rgdal,” “sp,” and “maptools” packages, and mapped output was produced in ArcGIS [Version 10.5.1].

## Results

### Regional impacts of climate change scenarios

Increases in temperature by region, from baseline, for the future climate scenarios, are synthesized in Table 2. Higher future temperatures are projected under all models and time periods evaluated for the continent.

**Table 2:** Average annual temperature increases (℃) from baseline (1960–1990) by region, RCP, and time period

Region	2030s		2050s		2080s	
	RCP 4.5	RCP 8.5	RCP 4.5	RCP 8.5	RCP 4.5	RCP 8.5
West Africa	1.32	1.57	2.29	2.32	2.84	4.38
East Africa	1.32	1.63	1.90	2.32	2.96	4.38
Central Africa	1.10	1.42	1.63	2.07	2.69	4.04
Southern Africa	0.94	1.28	1.33	2.01	2.51	4.08

### Current and future suitability risk

**Fig. 2:**
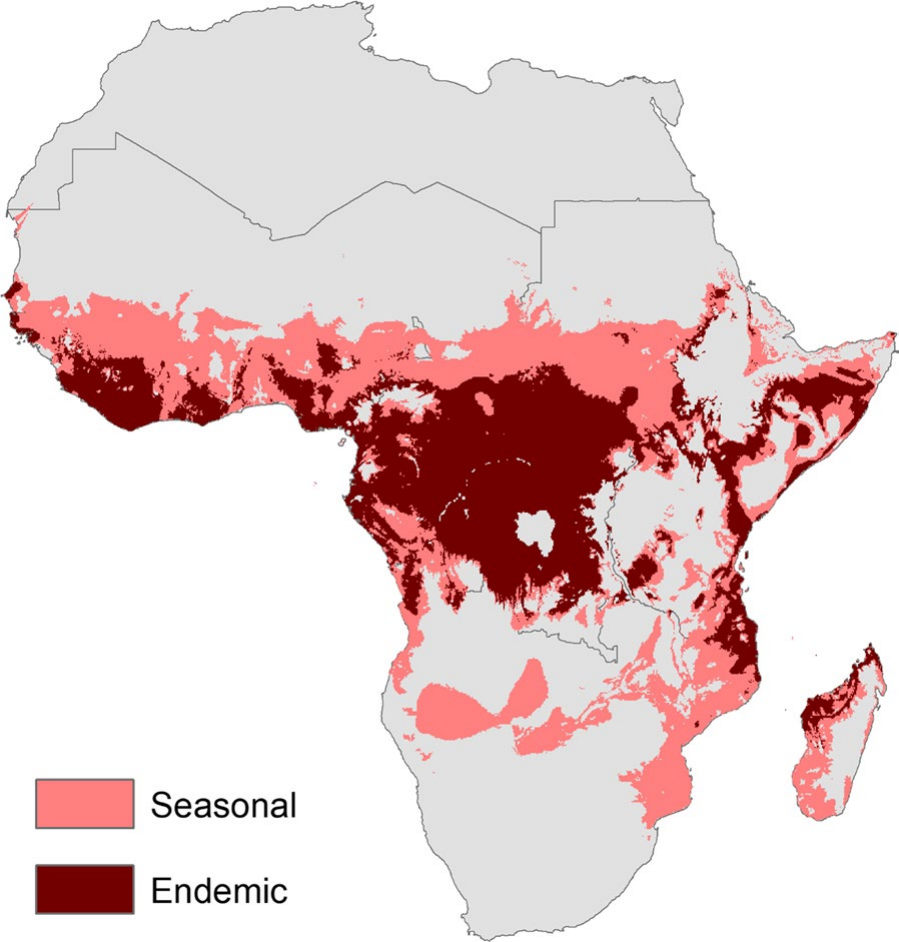
Modelled endemic (10–12 months) and seasonal (7–9 months) transmission suitability for malaria under current climate conditions

Under baseline conditions, the current distribution of predicted endemic (10–12 months) transmission suitability for malaria is concentrated in the Central African region, with additional areas along the southern coast of Western Africa, and along the eastern coast of Eastern Africa, and in the north of Madagascar (Fig. 2). Seasonal transmission (7–9 months of the year) suitability is predicted to occur along a band through Western and Eastern Africa, south of the areas too arid for mosquito life cycles, and in parts of Southern Africa, particularly through Mozambique.

**Fig. 3:**
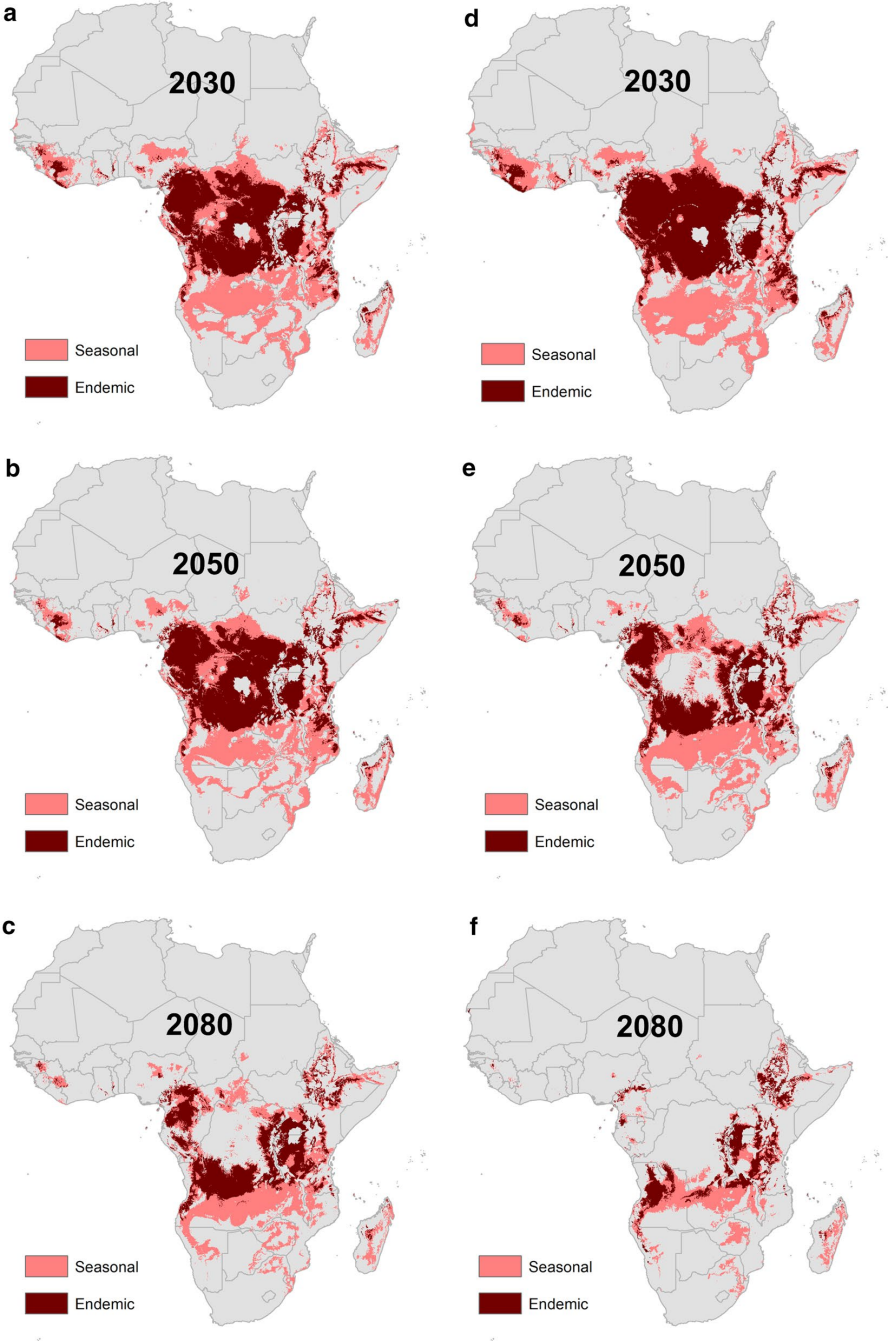
Modelled output of malaria transmission indicates shifting future endemic (dark red) and seasonal (light red) transmission suitability under two representative concentration pathways, RCP 4.5 (**a**–**c**) and RCP 8.5 (**d**–**f**), for the years 2030, 2050, and 2080

The projected future climate model impacts on malaria transmission suitability are shown for both RCP 4.5 and 8.5, for the three time horizons modelled, in Fig. 3. Hotspots of endemic suitability are predicted to emerge in the center of the continent, the East African highlands, the Lake Victoria region, and northern Zambia, becoming more pronounced in the latter part of the 21st century, under both the better (RCP 4.5) and worse (RCP 8.5) scenarios. A significant portion of these areas are located in Eastern Africa, including Uganda, Kenya, and Tanzania, a region with currently lower predicted suitability for endemic malaria transmission compared to Central and Western Africa. Additionally, areas predicted to have limited current suitability for *Anopheles* transmission may become seasonally suitable under conditions of a changing climate, including the Southern Africa region, which will see marked increases in areas predicted to be suitable for seasonal and endemic malaria transmission (Figs. 2 and 3).

Concentrated hotspots of predicted seasonal suitability appear in central Angola, northwestern Zambia, northern Tanzania, and the southern coast and northern part of Mozambique by 2030, in both climate scenarios (RCP 4.5 and RCP 8.5). This includes large portions of Zambia, Malawi, and Tanzania, eastern South Africa, Botswana, the highlands of Zimbabwe, northern Mozambique, and the Zambezi River Basin. Hotspots of seasonal malaria transmission suitability are predicted to either continue to concentrate or shift both northward and southward into the highlands of Ethiopia and Southern Africa toward the latter part of the 21st century.

### Shifting burden of transmission suitability—people at risk

**Fig. 4:**
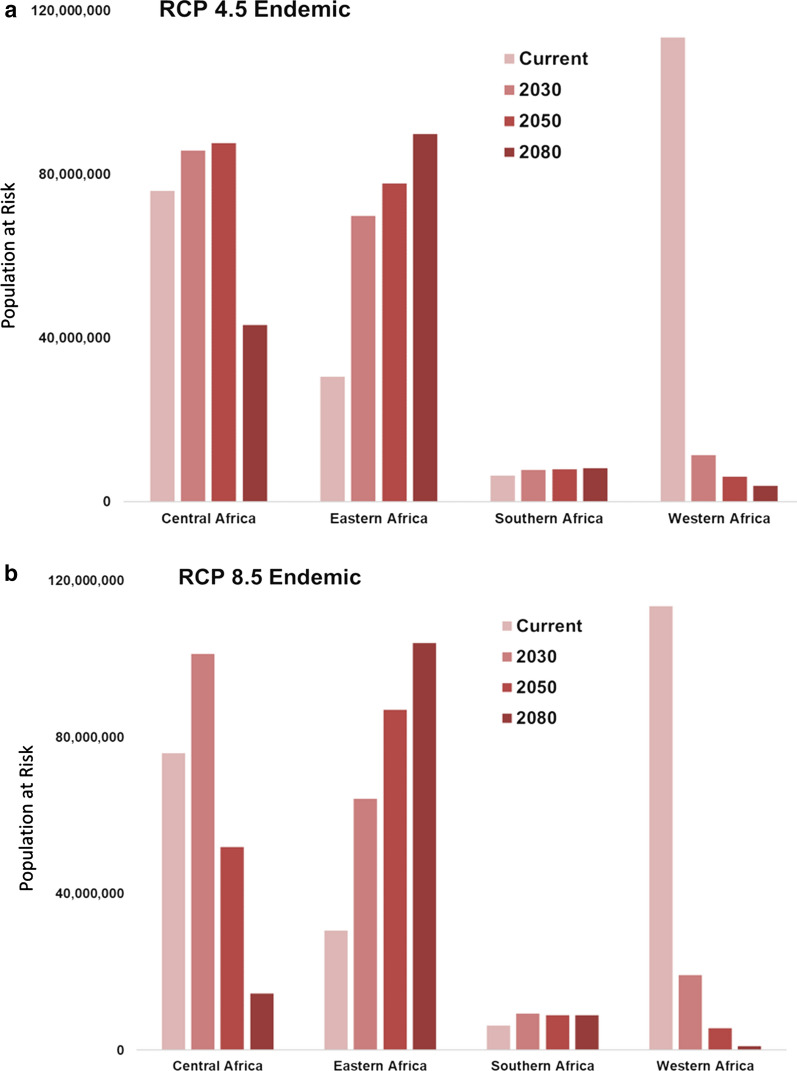
Population at risk (PAR) for exposure to endemic malaria transmission will change in the future as geographic suitability shifts under two scenarios of climate change, RCP 4.5 (**a**) and RCP 8.5. **b** Eastern Africa will regionally see dramatic increases PAR by the year 2080, while shifting suitability will largely relieve the burden of endemic transmission in Western Africa

**Fig. 5:**
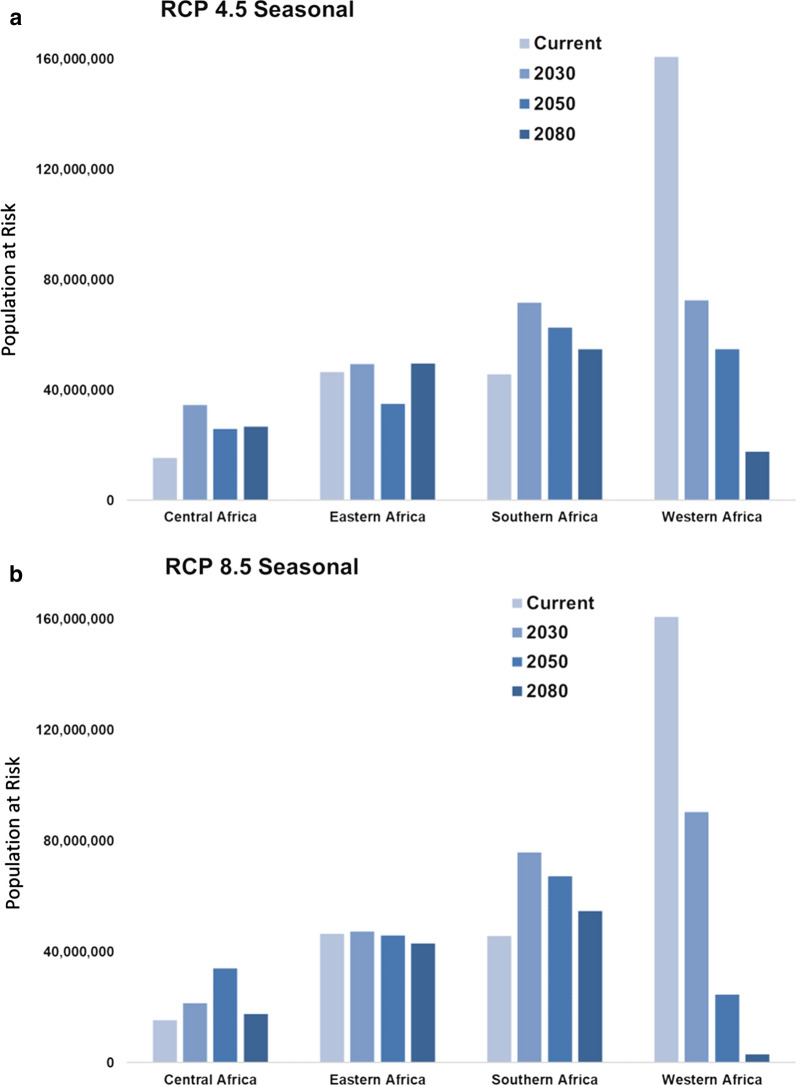
Population at risk (PAR) for exposure to seasonal malaria transmission will change in the future as geographic suitability shifts under two scenarios of climate change, RCP 4.5 (**a**) and RCP 8.5 (**b**). Southern Africa is predicted to have increased seasonal transmission, while shifting suitability will largely decrease seasonal transmission in Western Africa

Examining the results of projected climate transmission risk models—RCP 4.5 and RCP 8.5, across the 3 future time horizons of 2030, 2050, 2080—a low of 196 million and a high of 198 million people in Eastern and Southern Africa are predicted to be burdened with some degree of malaria transmission risk in the future due to shifting suitability by the 2080s. Regionally, by the year 2080, the worst-case scenario (RCP 8.5) predicts an additional 73.4 million people at risk from year-round exposure to transmission in Eastern Africa (Fig. 4). In spite of currently low endemic suitability, shifting seasonality in Southern Africa is predicted to place over 2.5 million additional people at risk for endemic transmission by the 2080s. In the short term, these changes are predicted to put the lives of a low of 50.6 million and a high of 62.1 additional people at increased risk for endemic transmission, and a low of 37.2 million and a high of 48.2 million people at risk for seasonal transmission, throughout Central, Eastern, and Southern Africa by the 2030s (Figs. 4 and 5). Given the strong empirical relationship between vector survival and temperature, as temperatures rise, exposure to malaria transmission is also expected to increase in previously unsuitable regions, such as those in the higher elevation regions of Southern and Eastern Africa. Countries predicted to be likely to be impacted by these changes include northern Angola, southern DRC, western Tanzania, and central Uganda in 2030; by 2080 these changes are predicted to extend into western Angola, the upper Zambezi River Basin, and northeastern Zambia, and will become more concentrated along the East African highlands.

These shifts in the geographic range of malaria suitability, broadly consistent across both scenarios of future climate, suggest both decreases and increases in the number of people exposed, depending on the climate scenario. The geographic and temporal evolution of future suitability of areas for malaria-transmitting *Anopheles* mosquitoes is closely tied to expected temperature changes under both RCP scenarios (Fig. 3). As temperatures rise, even within the next 12 years (by 2030), important changes are anticipated. Despite the dramatic projected reductions in endemic and seasonal malaria transmission risk in Western Africa (Figs. 4 and 5), shifting suitability due to climate change will still place additional people at risk. Taking moderate and marginal suitability for malaria transmission into account results in an overall projected net gain of 58.7 million (RCP 4.5) to 60.4 million (RCP 8.5) people who will experience some level of malaria risk in Western Africa by the 2030s. Large areas of coastal Western Africa and the Horn of Africa will likely exceed mosquitoes’ thermal tolerance, with suitability disappearing. At the same time, rising temperatures are predicted to increase the southern range of seasonal suitability for *Anopheles* mosquitoes into Southern and Central Africa, into western Tanzania. Under scenarios in the 2050s, both endemic and seasonal zones exhibit an eastward shift, with thermal threshold exceedance again apparent under the worst-case scenario (RCP 8.5), eliminating suitability across Central Africa. The end-of-the-century scenarios (2080) predict concentrated areas of endemism in previously unsuitable or marginally suitable areas, namely the highlands of East Africa and Southern Africa. Where the number of months of suitability for *Anopheles* survival decrease, opportunities will emerge to alter and define more targeted seasonal responses, either reducing the cost of interventions or providing a window into potential eradication to malaria exposure. Targets of opportunity include Central Africa (the Central African Republic, western Congo, Cameroon, and Equatorial Guinea) and coastal East Africa (Tanzania and Kenya).

### Novel endemic and seasonal risk

**Fig. 6:**
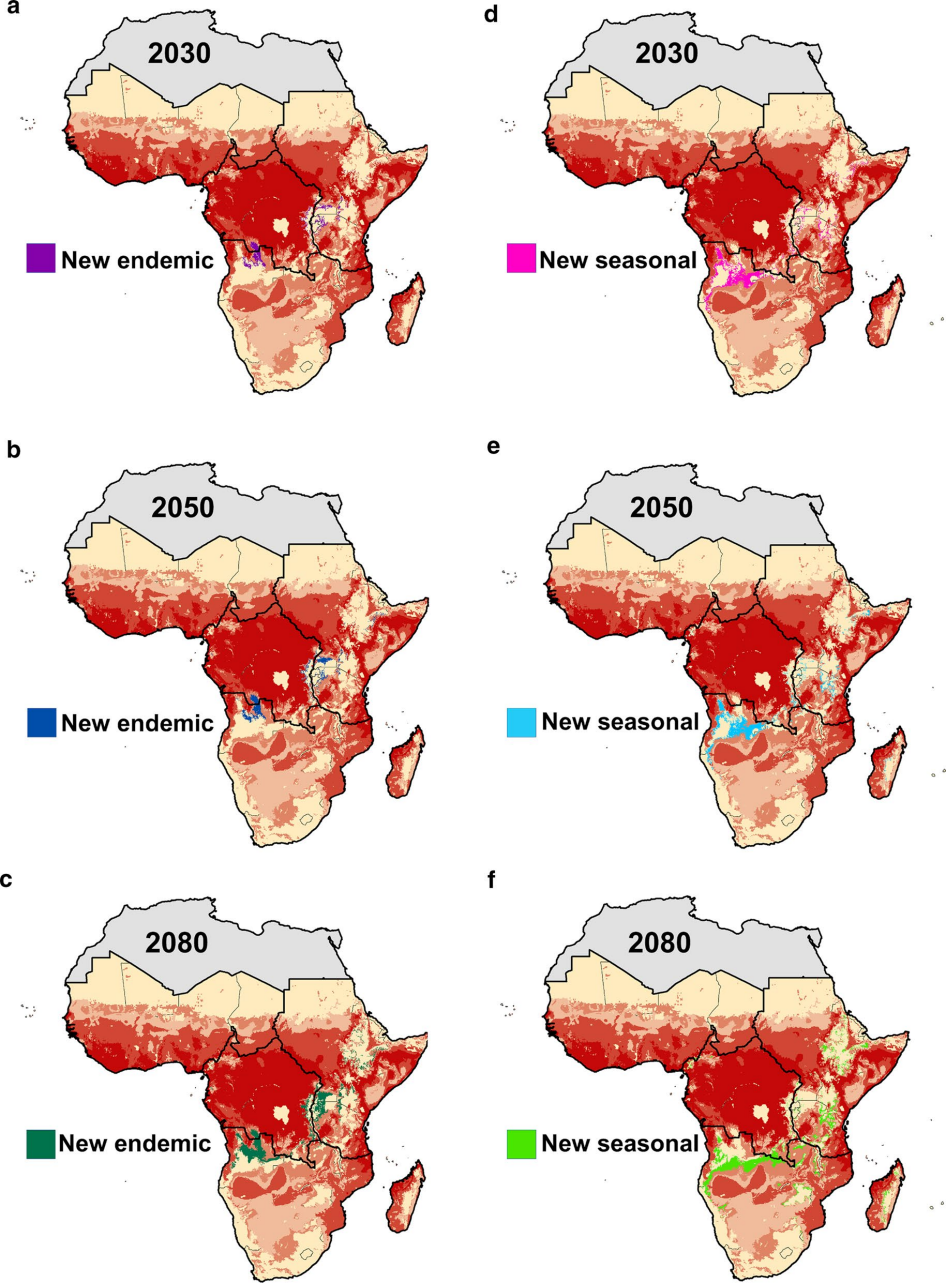
New areas of endemic (**a**–**c**) and seasonal (**d**–**f**) suitability, under RCP 4.5 for 2030, 2050, and 2080. Red shading intensity indicates current malaria suitability season

**Fig. 7:**
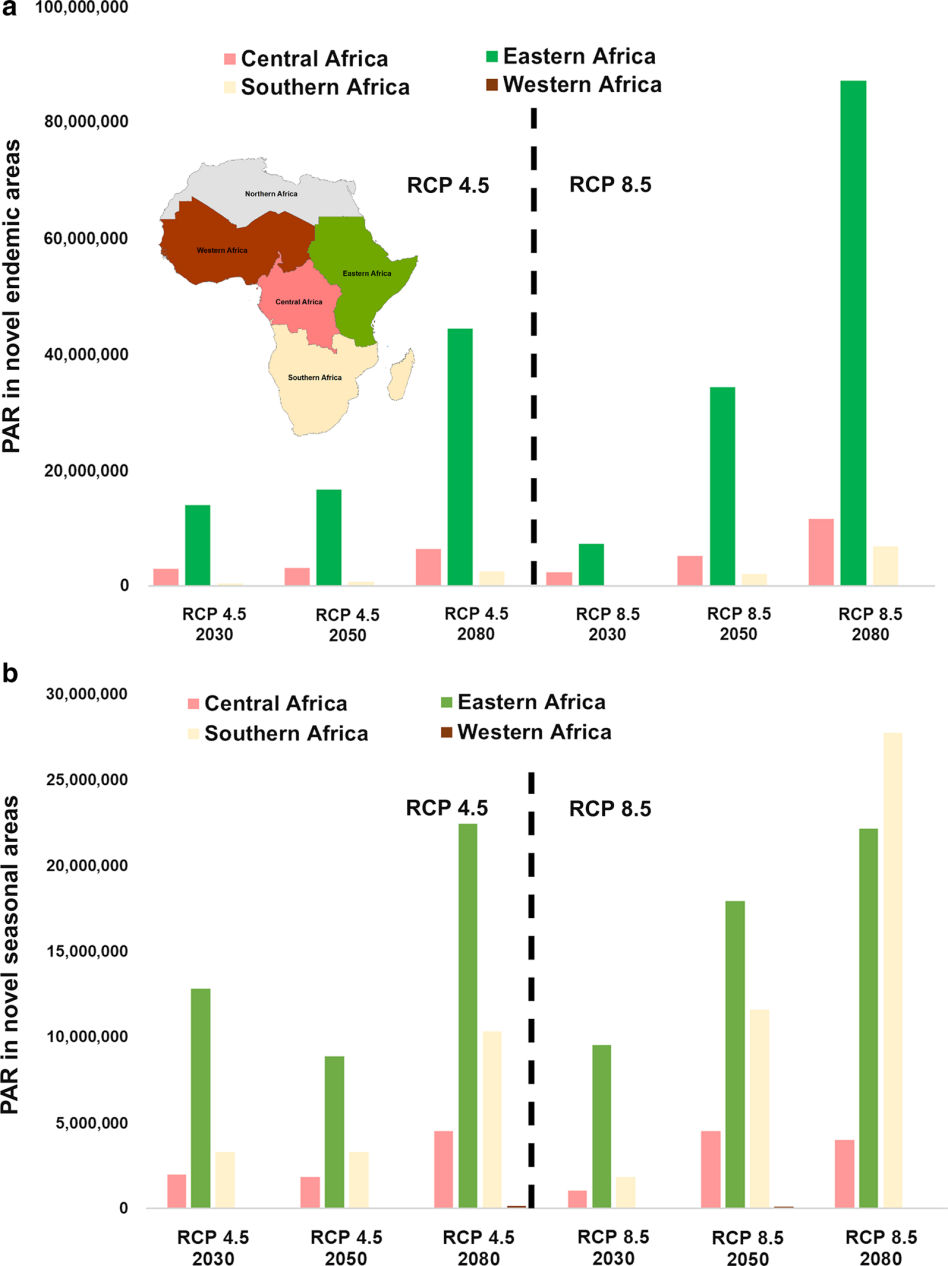
The number of people at risk (PAR) in **a** newly endemic (10–12 month) suitable areas, and **b** newly seasonal (7–9 month) suitable areas, for RCP 4.5 and RCP 8.5, in 2030, 2050, 2080

Some parts of sub-Saharan Africa currently predicted to experience no malaria transmission suitability risk will experience shifting suitability, resulting in novel areas with no history of malaria transmission becoming suitable for endemic and seasonal transmission in the future. As seen in Fig. 6, for RCP 4.5, this exposes populations along an arc extending into East Africa, leading to dramatic PAR increases for regional exposures, particularly novel endemic exposure increase in East Africa, and novel seasonal exposures in Southern Africa (Fig. 7).

## Discussion

The changes in the geographic range of malaria suitability, broadly consistent across both scenarios of future climate pathways modelled here, suggest that the number of people exposed to conditions of malaria suitability will both increase and decrease in sub-Saharan Africa, depending on the region. Thus, as some populations experience reduced burden of malaria risk in the future, shifting suitability will increasingly place naïve populations at risk for outbreaks, particularly in Southern and Central Africa. Malaria outbreaks that occur where people have little or no immunity to the disease can lead to epidemic conditions, especially among vulnerable groups such as women and children [1, 20]. This research identifies “hotspots” where current exposure and, therefore, immunity is nonexistent; these areas could see epidemic “flares” as climate conditions affect vector survival and reproduction. This effect may be further exacerbated in novel areas with no previous history of malaria exposure, where both immunity and knowledge regarding malaria prevention are lacking [21–23]. Malaria outbreaks occurring where people have acquired immunity due to prolonged and repeated malaria exposure trigger management actions employing a cadre of tools, including vector control and case management approaches to prevent or reduce transmission [23, 24].

These results identify regions where interventions need to be revisited to consider how climate will alter risk profiles in the future. The strong seasonal cycle of malaria across Southern Africa is related to climate and weather conditions [25, 26]. Thus, during some periods of the year, climate conditions are not conducive to spread of the disease. Given the strong empirical relationship between vector survival and temperature, as temperatures rise exposure to malaria transmission is expected to increase in previously unsuitable regions, such as those in the higher elevation regions of Southern and East Africa. A key concern with climate change impacts is whether climate change will lengthen the period of the year during which diseases can establish and be transmitted. For example, areas where spring and autumn are now too cold for the reproduction of malaria vectors may become more suitable in the future. In these areas, increases in temperature may not impact midsummer malaria incidence greatly, but may result in a longer season, extending into both spring and autumn, during which malaria incidences will occur. In some cases, malaria may shift from being a seasonal disease burden to a year-round burden. This will necessitate different types of management and control interventions than those currently in place for short-season malaria [27, 28]. Where the number of months of suitability for *Anopheles* survival decreases, opportunities will emerge to alter and define more targeted seasonal responses—either reducing the cost of interventions or providing a window into potential eradication to malaria exposure. An increase in the number of months where conditions are suitable for mosquito survival will require responses to be extended for longer periods of time, increasing resource needs (e.g. staff time, medicines) as well as costs [29]. In examining areas where malaria suitability is currently considered seasonally restricted, but will likely become more prevalent throughout the year, public health planners can anticipate which regions may require an extended investment pipeline.

A fundamental underpinning of modelling the response of vector-borne diseases to climate and ecology is the choice of model process. Previous approaches, such as that of the Malaria Atlas Project (MAP) and the Mapping Malaria Risk in Africa (MARA) project, are essentially top-down, wherein empirical data collected on the ground are matched to local climate conditions, and suitability established via geostatistical methods. In contrast, the modelling approach used here is mechanistic and “bottom-up,” wherein the life history of mosquitoes and pathogens, and their responses to temperature, are explicitly quantified based on empirical, laboratory-based data and incorporated into the model to predict where suitability for transmission is likely to occur. A mechanistic model, built independently of case outcome data, allows for validation with empirical, field-collected data, and obviates the bias of modelling data while intervention is ongoing, as is inevitably the case with previous approaches [30]. In this study, the upper quartile of a curve was the prediction space on temperature, so the model projection contains no description of uncertainty on the mechanistic side. Future explorations of this climate-risk modelling approach could examine the sensitivity of overall findings to either uncertainty bounds of the original model parameterization, or using a range of cutoffs (e.g. 20th and 30th percentile, to describe error range around the 25th percentile).

While substantial progress has been made in recent years in the provision and use of climate projections, considerable uncertainties remain with their use [31]. Using climate science research results to inform the decision process about which policies or specific measures are needed to tackle climate impacts requires acknowledging the uncertainties inherent in climate projections. These uncertainties may arise from mathematical reductions (parameterizations) of climate phenomena; potential socioeconomic technological pathways and attendant carbon cycle feedbacks that influence atmospheric concentrations of key greenhouse gases; imperfect scientific knowledge and the computational constraints of modelling regional detail while still incorporating relevant large-scale climate patterns; and the relationship between climate models and their relative impacts on key sectors and resources [31–33]. Furthermore, uncertainty can arise over the chance of a single event (for example, crossing a threshold), recurrent events (the return period of a flood, for example), discrete events (hurricane frequency), and complex events (for example, the interplay of different factors that lead to drought) [34]. Recognizing this, good practice is followed by incorporating a multimodel range of climate projections rather than a single model, as performed in this study [31, 35, 36]. However, as with the mechanistic transmission model, using a multimodel climate ensemble does not yield an expression of uncertainty bounds. This study is a snapshot of model output at three time horizons: 2030, 2050, 2080, and two future scenarios, in terms of representative concentration pathways (RCPs), one ‘better’ (4.5) and one ‘worse’ (8.5), to give an estimate of potential future values in, rather than picking one scenario. As these RCPs are predicated on potential future cases of mitigation, it is assumed that the future will unfold within this range of possibility, at present there are no grounds to put more credence in one or the other. In future exploration of this modelling approach, unpacking the range of estimated risk (geographic and people) generated under the different models in the ensembled product, and exploring the 4 RCPs currently considered in IPCC projections, would give a richer view of the range of future possibility.

For the population data specifically, it is important to recognize that the projected population for 2020 is used to calculate the numbers of people potentially affected by changing suitability conditions across all future time periods. As with climate models, these projections do not necessarily capture all the factors that drive population movement and growth, and should be taken as best modelled estimates rather than exact values. Future studies can incorporate the additional projected population responses to climate change scenarios themselves, via such projects as the Shared Socioeconomic Pathways (SSP) projections, which offer multiple scenarios of population changes in response to potential social, environmental, and other drivers of societal development and change, as the climate changes into the future [37]. Given the range of optimal transmission suitability temperatures for malaria, which corresponds with likely optimal human conditions, this study may, unfortunately, be underestimating future vulnerable populations, as people move into areas more suitable for transmission risk.

The study results are based on the temperature response curves of both *Anopheles* mosquitoes and malaria pathogens. Nevertheless, many studies point to the critical role that rainfall plays in vector survival across sub-Saharan Africa [12, 14, 15]. For example, single, intense rainfall events can wash away critical breeding sites, leading to a reduction in transmission potential [16, 38]. Similarly, too little rainfall can limit mosquito survival as moisture is a prerequisite for breeding habitat [39]. The approach herein addresses this second issue by masking out areas that are too arid for mosquito survival. While the relationship between rainfall and *Anopheles* survival is critical, the available projections of rainfall are uncertain at the geographic scale of this work and, therefore, are not considered in this analysis.

Geographically projected model outputs are a useful component of a planning and intervention framework, providing a means of communicating key areas of risk and affected populations to decision makers. Anticipation of not only the location and time, but the duration of potential outbreak events will facilitate the development of efficient and timely agency responses. Moreover, this framework serves as a foundation for scenario analysis, explicitly modelling risk of exposure for different climate scenarios and time horizons. The range of potential outcomes allows governments and agencies the flexibility needed to reasonably anticipate resource use and funding needs, enabling the development of adaptive intervention strategies for both near and long-term outcomes.

## Conclusions

Addressing the changing risk profiles projected in this suitability analysis will require modifying current interventions and programmes and implementing new ones to explicitly consider climate variability and change. While these projections of mechanistic models coupled to ensembled climate predictions for fixed time horizons (2030, 2050, 2080) and fixed population projections are simplified, they present a framework within which those potential futures can be explored, and examine both where and when changing risk is anticipated. Opportunities for improved responses also exist, including detailed geographic targeting, optimizing strategies and seasonal alignment with interventions. Identifying high risks in new areas of suitability present opportunities for informed action. Where malaria suitability is currently nonexistent to newly suitable, whether seasonal or endemic, the risks are critical, especially given that local populations’ immunity will be low. This could lead to the potential emergence of novel strains, rapid resistance, and untimely identification, translating into epidemic outbreaks. To respond, targeted and informed geographic surveillance in these regions could help to prepare timely responses before epidemic outbreaks occur. Knowing where and when more people will potentially be exposed offers an opportunity to increase the investment timeframe (seasonal to year-round), optimize vector control, and improve case management, with the evidence base to support these actions. Moving down the path toward elimination for some regions, where malaria transmission suitability decreases, opportunities will arise to focus resources on making surveillance and response systems increasingly sensitive and focused to identify, track, and respond to malaria cases and any remaining transmission foci.

## Data Availability

Data sharing is not applicable to this article as no datasets were generated or analysed during the current study.

## Abbreviations

MAP: Malaria Atlas Project
MARA: Mapping Malaria Risk in Africa
NDVI: Normalized difference vegetation index
GCM: Global climate model
CMIP5: Coupled Model Intercomparison Project
CF: Change factor
RCP: Representative concentration pathway
PAR: Population at risk
GPW: Gridded Population of the World
