# Correction: Shifting transmission risk for malaria in Africa with climate change: a framework for planning and intervention

**DOI:** 10.1186/s12936-023-04715-y

**Published:** 2023-09-25

**Authors:** Sadie J. Ryan, Catherine A. Lippi, Fernanda Zermoglio

**Affiliations:** 1 Emerging Pathogens Institute, University of Florida, Gainesville, FL USA; 2 Department of Geography, University of Florida, Gainesville, FL USA; 3 College of Agriculture, Engineering, and Science, University of KwaZulu-Natal, Durban, South Africa; 4 Chemonics International, Washington, DC USA

**Correction: Malaria Journal (2020) 19:170** **https://doi.org/10.1186/s12936-020-03224-6**

Following publication of the original article [1], the authors requested to correct a sentence in the second paragraph of the Results subsection ‘Shifting burden of transmission suitability—people at risk’ to clarify their intended meaning. Namely, the following sentence has been corrected:

- “Shifting suitability due to climate change will place additional people at risk despite reductions in endemic and seasonal malaria transmission, resulting in a net gain of a low of 58.7 million and a high of 60.4 million people who experience some level of malaria risk in Western Africa by the 2030s.”

The sentence has been replaced with the following:

- “Despite the dramatic projected reductions in endemic and seasonal malaria transmission risk in Western Africa (Figs. 4 and 5), shifting suitability due to climate change will still place additional people at risk. Taking moderate and marginal suitability for malaria transmission into account results in an overall projected net gain of 58.7 million (RCP 4.5) to 60.4 million (RCP 8.5) people who will experience some level of malaria risk in Western Africa by the 2030s.”

The authors thank you for reading this erratum and apologize for any inconvenience caused.
